# Effects of Social Isolation and Loneliness in Children with Neurodevelopmental Disabilities: A Scoping Review

**DOI:** 10.3390/brainsci10110786

**Published:** 2020-10-28

**Authors:** Celia Kwan, Mojgan Gitimoghaddam, Jean-Paul Collet

**Affiliations:** 1 Department of Pediatrics, Faculty of Medicine, University of British Columbia, Vancouver, BC V6T 1Z4, Canada; celia.kwan@alumni.ubc.ca (C.K.); mgitimoghaddam@bcchr.ca (M.G.); 2 BC Children’s Hospital Research Institute, Vancouver, BC V5Z 4H4, Canada

**Keywords:** pediatrics, loneliness, isolation, neurodisability, children

## Abstract

Loneliness and social isolation have negative consequences on physical and mental health in both adult and pediatric populations. Children with neurodevelopmental disabilities (NDD) are often excluded and experience more loneliness than their typically developing peers. This scoping review aims to identify the type of studies conducted in children with NDD to determine the effects of loneliness and/or social isolation. Three electronic databases (Ovid MEDLINE, EMBASE, PsychINFO) were searched from inception until 5 February 2019. Two independent reviewers screened the citations for inclusion and extracted data from the included articles. Quantitative (i.e., frequency analysis) and qualitative analyses (i.e., content analysis) were completed. From our search, 5768 citations were screened, 29 were read in full, and 12 were included. Ten were case-control comparisons with cross-sectional assessment of various outcomes, which limited inference. Autism spectrum disorder, attention-deficit/hyperactivity disorder, and learning disorder were the most commonly studied NDD. This review showed that loneliness among children with NDD was associated with negative consequences on mental health, behaviour, and psychosocial/emotional development, with a likely long-term impact in adulthood. Lack of research in this area suggests that loneliness is not yet considered a problem in children with NDD. More studies are warranted using prospective designs and a larger sample size with a focus on the dynamic aspect of loneliness development.

## 1. Introduction

Belonging is a basic human need—a psychological “need to love and care for others, and to believe we are loved and cared for in relationships” [1]. This need can be explained by a common human goal: to stay alive. According to terror management theory, self-preservation is the ultimate goal toward which all behaviour is directed [2]. Hence, we are motivated to seek social attachments, just like we strive to maintain an appropriate temperature and blood sugar level, so that we can stay alive and increase our chances of gene perpetuation [2].

Social interaction is thus a crucial component in human experiences. A lack of this interaction can lead to social isolation (defined by a decrease of social contacts) and even to the feeling of loneliness. There is currently no widescale consensus on the definition of loneliness. Bolmsjo et al. define existential loneliness as “the immediate awareness of being fundamentally separated from other people and from the universe, primarily through experiencing oneself as mortal, or, and especially when in a crisis, experiencing not being met (communicated with) at a deep human (i.e., authentic) level, and typically therefore experiencing negative feelings, that is, emotions or moods, such as sadness, hopelessness, grief, meaninglessness or anguish” [3]. Meanwhile, Weiss describes emotional loneliness as “the lack of a close, intimate attachment to another person”, giving rise to feelings of emptiness and anxiety, and social loneliness as “the lack of a network of social relationships in which the person is part of a group of friends who share common interests and activities.” [4]. Despite the lack of consensus on the definition of loneliness, many agree that it is a “subjective negative experience that results from inadequate meaningful connections” [5,6,7,8,9]. It is important to note that the subjective perception of loneliness can be different than the objective assessment of social isolation; loneliness can be synonymous with a painful perception of social isolation [5].

Loneliness can have many consequences on an individual’s health, both physically and mentally. Hawkley and Cacioppo published a review of the literature on adult loneliness in 2010 and discussed the impact of loneliness on mental health and cognitive functions [5]. They reported that loneliness has been associated with increased depressive symptoms, suicide, anxiety, and anger. They also found that loneliness was associated with increased perceived stress while diminishing one’s optimism and self-esteem [5].

Similarly, in the pediatric population, the distressing feeling of social isolation has consequences on children’s mental health. For instance, in a 2018 cross-sectional study conducted with 5925 children and adolescents from grades 5 (mean age = 11.8 years), 7 (mean age = 13.8), and 9 (mean age = 15.8), Lyyra et al. found loneliness to be statistically significantly (*p* < 0.001) correlated (Pearson’s) with many somatic and psychological symptoms such as headache (r = 0.23), stomach ache (r = 0.23), anxiety (r = 0.32), and feeling low (r = 0.5) [6]. Similarly, in an international cross-sectional study conducted in 2016, Stickley et al. found that lonely adolescents had higher odds of experiencing anxiety and depressive symptoms as well as somatic symptoms like headache or nausea [10].

Two excellent reviews of the literature on loneliness in the adult population report consequences on physical health [5,11]. In 2015, one review of 33 articles reported an association between loneliness and many chronic conditions, such as heart disease, hypertension, stroke, lung disease, and metabolic disorders [11]. Moreover, for cardiovascular diseases, loneliness was also associated with hospital readmissions, longer lengths of stay, overutilization of healthcare resources, and increased odds of death [11]. Likewise, Hawkley and Cacioppo reported that loneliness was associated with increased cardiovascular health risk and mortality [5].

It is important to note that these negative outcomes associated with loneliness/social isolation can persist many years down the road. In a 2006 longitudinal study done by Caspi et al. which followed 1037 children from birth to age 26, it was shown that compared to non-isolated children, socially isolated children were at significant risk of poor adult health [12]. Similarly, in 2007, Wilson et al. identified that among 823 initially dementia-free older adults, those with a higher degree of loneliness (as measured by the de Jong-Gierveld Loneliness Scale) were 2.1 times more likely to develop Alzheimer’s disease within the next four years when compared to those with a lower degree of loneliness [13].

Unfortunately, despite the importance of social interactions and subsequent early learning and development, children with neurodevelopmental disabilities (NDD) are often excluded in our society because of their functional and behavioural limitations [14]. According to the Diagnostic and Statistical Manual of Mental Disorders (DSM) 5, NDD “are characterized by developmental deficits that produce impairments of personal, social, academic, or occupational functioning” [15]. This definition includes, for example, autism spectrum disorder (ASD), attention-deficit/hyperactivity disorder (ADHD), specific learning disorder, developmental coordination disorder, or tic disorder [15]. The behaviour of children with NDD may not always conform to social norms and they are thus at a higher risk for social exclusion, with possible subsequent feelings of loneliness [16]. In fact, several studies found that children and adolescents with autism were lonelier compared to typically developing children [17,18,19,20,21]. In a 2009 case-control study involving 39 adolescent boys with ASD and 199 boys without ASD, Lasgaard et al. found that ASD was associated with “often or always” feeling lonely (OR of 7.08, *p* < 0.0005) [20]. Similarly, in studies done on boys with developmental coordination disorder (DCD) and on adolescents with a learning disorder (LD), it was shown that these populations reported significantly more loneliness compared to the control group without NDD [22,23,24]. For instance, in 2007, Poulsen et al. found that boys with DCD had statistically significant higher feelings of loneliness compared to controls (F (3.169) = 45.61, *p* < 0.001) [22]. However, it is important to note that there is a wide range of socio-emotional deficits across the different NDD; even within one NDD (such as ASD), there can be variability in the deficits and also various degrees of severity in the expression. The experience of social exclusion and loneliness may, therefore, vary significantly depending on the child’s level of socioemotional deficits, as well as other factors such as intelligence, language ability, disorder severity, chronological age, and environmental context.

Feelings of loneliness early in life may affect child development and learning abilities, in addition to the physical and mental health consequences mentioned above. As per Vygotsky’s model of sociocultural learning, the development of mental processes in children is determined by their relationship within a positive social environment [25]. Children acquire tools such as language, signs, and symbols as they communicate with adults or more experienced peers [25]. In a review of literature, Parker et al. identified the importance of social acceptance for psychosocial development and presentation of depression or school drop-out [26]. Social isolation from exclusion can thus limit a child’s opportunity to interact with their social environment and as a result, delay their development and learning of social norms. Children with NDD (primary disability) who suffer from social isolation/loneliness can thus experience a “secondary disability” due to the consequences of loneliness on their health and development [27].

Although there have been reviews completed on the effects of social isolation/loneliness in the adult population [28], to our knowledge, there has not been a review conducted to synthesize the available literature of these effects in children with NDD specifically, even if they are at higher risk of social exclusion. Moreover, the available literature focusing on loneliness/social isolation in children with NDD seems to be quite scarce compared to that in the general pediatric population. Given the universal need for belonging and the potential health consequences that can arise from social isolation and loneliness, we felt the need to synthesize the current literature specifically on children with NDD. This may not only help us better understand the impact of loneliness and social isolation in this particular population, but can also serve as a rationale for future projects aimed at mitigating loneliness in this group.

The objective of this scoping review of the literature is to, therefore, investigate what has been published on the association between social isolation and/or loneliness and the health and development of children with NDD. Since this is the first review on this topic, a scoping review was conducted to help map the literature as well as identify the populations studied, the study designs, the outcomes and key concepts, and the gaps in the present literature. Given that our review topic is already quite broad, we will not be addressing the development process of loneliness in children with NDD or the types of interventions available to prevent or decrease loneliness. At this point, we felt that it was more appropriate as a first step to explore the studies conducted and the types of outcomes studied.

## 2. Materials and Methods

This scoping review was undertaken utilizing the scoping review methodological framework by Arksey and O’Malley [29] and the guidance for the conduct of scoping reviews by the Joanna Briggs Institute [30].

### 2.1. Eligibility Criteria

The PICO (Population, Interest, Context) framework was used to guide the selection of articles: our research question was “how does social isolation or loneliness (exposure) affect the health or development (outcomes) of children and adolescents (0–18 years of age) with NDD (population)”. A comparison group was not specified to keep the research question broad, since this is a scoping review. Studies were included if they met the following inclusion criteria: (a) The study was published in English or French; (b) primary focus on children and adolescents (0–18 years of age) with NDD; (c) the study investigated the link between loneliness/social isolation and child health or development. All study designs were eligible.

### 2.2. Search Strategy:

An electronic search was conducted by one author (C.K.) with the assistance of an academic librarian of the following databases from inception until 5 February 2019: Ovid MEDLINE, Ovid EMBASE, and PsychInfo. These databases were selected after a consultation with the librarian and the research team because they were the most relevant to the field of our research question. The complete search for each database is documented in Appendix A, including the keywords used. These keywords were chosen with the help of the librarian and the research team and they relate to NDD (e.g., autism, learning disorder) and loneliness or social isolation (Appendix A). Specifically, for NDD, we searched the MeSH “Neurodevelopmental Disorders” and exploded it to include all the narrower terms under it. We also searched all of those narrower terms as keywords. Since Down Syndrome and Cerebral Palsy were not included under the NDD MeSH, those were searched as both MeSH and keywords as well. There were no restrictions in the year of publication or in the study design. We did not search in grey literature. A hand search of the reference lists of all included papers was done by two authors (C.K. and J.P.C.) to reveal more articles.

### 2.3. Study Selection/Screening:

All articles that resulted from the search process were imported into the EndNote database system. Duplicates were removed. Using the study inclusion/exclusion criteria, two reviewers (C.K. and J.P.C.) independently screened the articles’ titles and identified which abstracts should be reviewed. This step led to the identification of articles for full-text review, performed by two reviewers independently. In case of discrepancies in the assessment and divergence of opinion, the controversial point was discussed and resolved with a third investigator (M.G.) with expertise in the area.

### 2.4. Data Collection and Synthesis:

A data extraction table (Table 1) was created, reviewed, and finalized by all authors. Two reviewers (C.K. and J.P.C.) extracted the following data from the included articles: Author (year), Study Title, Country of Origin, Study Design, Sample size, Participants characteristics (age, gender, diagnosis), Study objectives, Outcomes assessed, Measurement tools, Key results of the study, and Study limitations.

The synthesis of the included articles is composed of a quantitative analysis component (e.g., frequency analysis) and a qualitative analysis component (i.e., content analysis). Only statistically significant correlations (*p* < 0.05) are reported in our review, except in the case where the study did not find any significant results—then, their non-significant results are reported.

### 2.5. Methodological Quality Appraisal:

We critically appraised the methodological quality of the included articles using the Quality Assessment Tool for Studies with Diverse Designs (QATSDD), which is a 16-item quality assessment tool that can be applied to various study designs [31]. This tool has been shown to have good reliability and validity (Kappa = 71.5%) [31].

## 3. Results

### 3.1. Identified Studies

**Figure 1:**
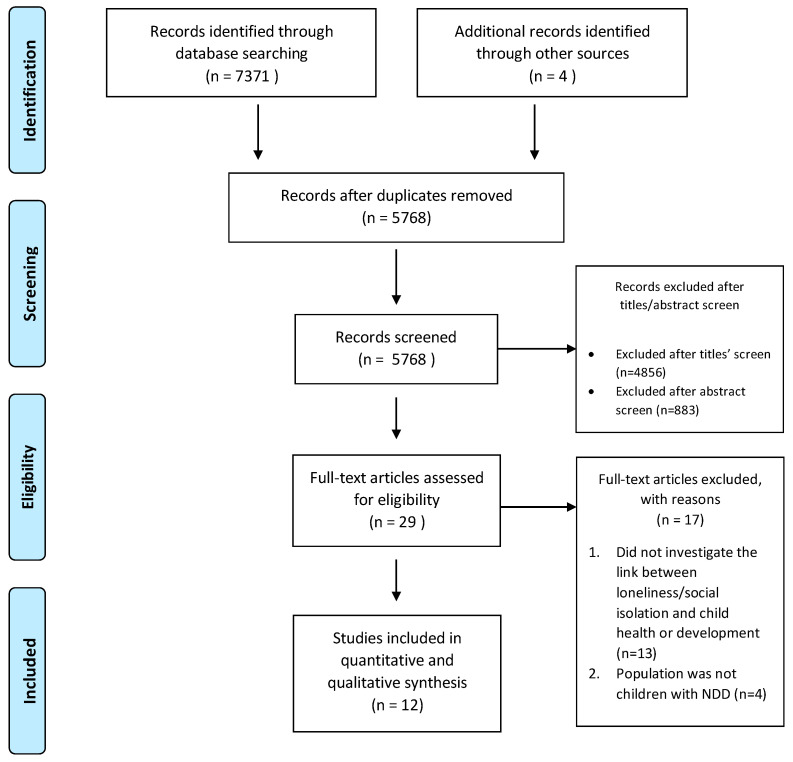
PRISMA flow diagram detailing the database searches, the number of abstracts screened, and the full texts retrieved.

Figure 1 shows the number of articles identified at each stage of the search process. A total of 7375 articles resulted from our initial search: 7371 from the databases and 4 from the reference lists of included articles. Of the 7375 articles retrieved, there were 5768 articles after removing duplicates. After doing a title screen, 912 articles were included for an abstract screen. After the abstract screen, 29 articles were included for a full text review. Then, 17 of those 29 articles were removed; 13 of the 17 did not investigate the effects of loneliness/social isolation on child health or development and 4 of the 17 did not involve children with NDD. In the end, our scoping review included 12 articles. They are summarized in Table 1 and are grouped by diagnoses of NDD and age groups. The first five rows of the table include the diagnosis of LD and the following rows consist of an ADHD diagnosis and finally, ASD. Within each NDD diagnosis, the studies are presented by chronological age of the study participants, with the younger age groups presented first and the older participants presented later.

### 3.2. Study Characteristics

Studies were published between 1999 and 2019, but the majority (*n* = 11, 92%) after 2007. Most studies were conducted in the United States (*n* = 5). Other studies were from Israel (*n* = 4), Australia (*n* = 2), and Norway (*n* = 1). Most studies were cross-sectional or case-control of prevalent cases with cross-sectional assessment of the youth’s characteristics (*n* = 10) [24,32,33,34,35,36,38,39,40,42] and only two studies had a longitudinal methodology with prospective follow-up [41,43].

### 3.3. Study Participants

Sample sizes varied from 20 to 1434 (median of 157). Four of the 12 studies had a sample size less than 100 participants (Table 1). The age range of participants in the included studies varied from 1 to 18 for 10 studies. Two studies included adolescents and adults as participants [41,42]. We decided to keep them because one study controlled for age and did not find that the pattern of results was affected [42] and the other study included mostly youth aged 17–18 (>50%), with few young adults (maximum age of 22). [41] Most studies (*n* = 6) included both children and adolescents [32,33,34,38,39,40], with adolescents defined as 13 years and up. One study only included children as participants [36], two studies only included adolescents [24,35], and two included adolescents and adults [41,42], with adults being defined as older than 18 years. One study followed children from age 1 to age 23 [43].

Amongst the NDD diagnoses in the included studies, ASD (including Asperger’s syndrome) was studied in five articles [38,39,40,41,42]. Similarly, five studies included learning disabilities (LD) [24,32,33,34,35]. Other diagnoses included: ADHD in two studies [24,36], comorbid LD and ADHD (*n* = 1) [24], Down Syndrome (*n* = 1) [43], developmental delay of unknown etiology (*n* = 1) [43], and motor impairment (*n* = 1) [43]. The study that included the diagnosis of motor impairment used data from the Early Intervention Collaborative Study, where children with motor impairment were defined as having “evidence of abnormal muscle tone or a coordination deficit along with delayed or deviant motor development, with or without other delays” [44].

### 3.4. Measurement of Loneliness and Social Isolation

Ten of the 12 included studies investigated loneliness in children with NDD. To measure loneliness, seven of 10 studies used the Loneliness and Social Dissatisfaction Scale by Asher et al. or its adaptation [32,33,34,35,36,39,43]. This is a 24-item questionnaire that can be completed by children, with the aim to assess children’s feelings of loneliness and social dissatisfaction [37]. There are 16 primary items that focus on children’s feeling of loneliness, social adequacy vs. inadequacy, or subjective estimations of peer status. The other eight items are “fillers” that focus on their hobbies to make them feel more open and relaxed [37]. This scale has been found to be internally consistent (Cronbach’s alpha = 0.90) and internally reliable (Spearman-Brown reliability coefficient = 0.91) [37]. A recent study in Turkey found this scale to have a test-retest reliability of 0.83 (Pearson’s correlation) [45]. Other scales used to measure loneliness included: the Peer-Network Loneliness and Peer-Dyadic Loneliness Scale (*n* = 1) [24], the University of California Los Angeles Loneliness Scale-Short Form (*n* = 1) [42], and the De Jong-Gierveld Loneliness Scale (*n* = 1) [40]. These scales have also good psychometric properties, but they have not been used as often as Asher’s scale.

Only three of the 12 studies investigated social isolation in children with NDD, including one study that investigated both loneliness and social isolation [36]. One study used the Child Behavior Scale [36] and another used the Child Behaviour Checklist [38] to measure peer isolation/friendship. The Child Behavior Scale is widely used in pediatrics. It is a teacher report measure that has a 6-item “Asocial with Peers” scale and a 7-item “Excluded by Peers” scale to measure peer withdrawal and peer exclusion, respectively [36,46]. Each item has a 3-point scale (doesn’t apply, applies sometimes, certainly applies). Both validity and reliability properties are excellent [47] and internal consistency is high (Cronbach’s alpha = 0.87–0.96) [36,46]. Similarly, the Child Behaviour Checklist is one of the most widely accepted rating scales used to assess emotional, behavioural, and social problems. It is completed by parents for children aged 6–18 years and parents report on the child’s friendships and participation in sports, hobbies, and clubs [38,48]. It also has good psychometric properties (both validity and reliability), with a Cronbach’s alpha of 0.91 [49]. Finally, one study used questions from the National Survey of Families and Households, modified to be appropriate for adolescents and adults, to measure social participation [41].

### 3.5. Study Outcomes

Table 1 summarizes the main outcomes studied in the reviewed articles. Amongst the 12 studies, only three examined social isolation. Two of these three focused on behavioural expression and had contradictory results [38,41]. In Lounds Taylor, Adams, and Bishop’s study, social participation was negatively correlated with internalizing symptoms in adolescents with ASD, with a correlation coefficient of −0.26 [41]. However, in the Dovgan and Mazurek’s study (2019), the number of friends was not significantly correlated with internalizing problems in youth with ASD [38]. The other study on social isolation and mental health outcomes was from Becker [36]. This study in 2015 did not find a significant correlation between peer exclusion and depressive symptoms or anxiety in children with ADHD, but did find a positive correlation with Oppositional-defiant disorder (ODD)/Conduct Disorder (CD) symptoms (r = 0.26, *p* = 0.005) [36].

**Figure 2:**
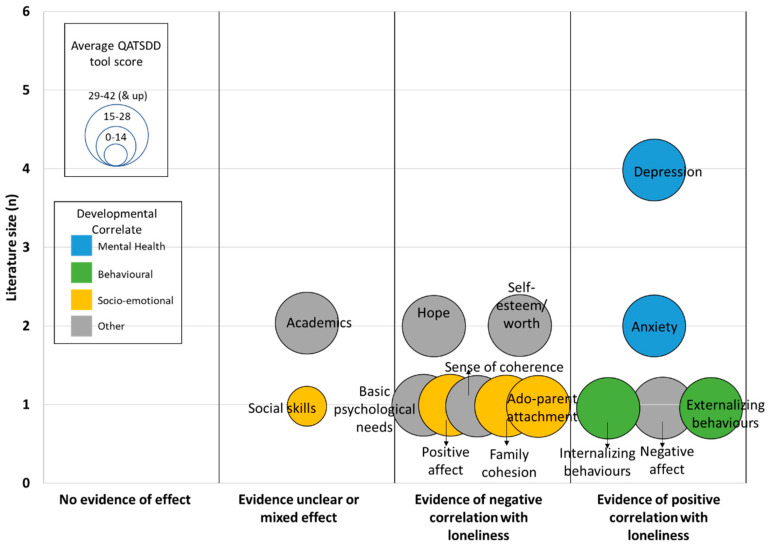
Evidence map of the literature on the effects of loneliness in children with neurodevelopmental disabilities (NDD).

The remaining articles focused on loneliness (*n* = 10). Amongst these 10, many associations between loneliness and child health or development were investigated and could be grouped into the following three categories: mental health, child development (behavioural, socio-emotional), and other outcomes. Figure 2 provides an evidence map of these 10 studies. Of note, none of the studies investigated the impact of loneliness/social isolation on physical health outcomes.

With regards to mental health, four studies investigated the link between loneliness and depression in children with NDD [33,40,42,43] and they all found a positive correlation. In 2018, Hedley et al. found that loneliness was positively correlated with depression in individuals with ASD (adolescents and adults), with a Pearson’s bootstrapped correlation of 0.44 (*p* < 0.01) [42]. Likewise, in 1999, Valas showed a significant positive correlation (Pearson correlation of 0.31, *p* < 0.01) between loneliness and depression in their sample of students [33]. In a prospective study, in 2013, Tillinger et al. found that age 10 loneliness in those with developmental disabilities could predict depressive symptoms at age 18, with a beta of 0.817 (*p* = 0.013); they also found a strong correlation between higher levels of loneliness at age 10 and higher levels of depressive symptoms at age 18 [43]. Finally, in 2009, Whitehouse et al. explained that loneliness in adolescents with Asperger’s syndrome was a significant mediator between the conflict/betrayal subscale of the friendship quality questionnaire and levels of depressive symptoms [40].

Two studies investigated the link between loneliness and anxiety in children with NDD and they both found a positive association. In Becker’s study, anxiety was positively associated (β = 0.62, *p* < 0.001) with loneliness in children with ADHD [36]. Similarly, White and Roberson-Nay’s study in 2009 showed a significant positive correlation between social anxiety and global loneliness in youth with ASD (r = 0.50, *p* = 0.04) [39]. Finally, Becker found in children with ADHD that perceived social acceptance was negatively associated with anxiety (β = −0.38, *p* < 0.001) [36].

Internationalizing and externalizing behaviours were studied by Al-Yagon et al. [24]. They found a statistically significant correlation between loneliness and internalizing behaviours in adolescents with LD (Table 1). They also found a significant correlation between peer-network loneliness and internalizing behaviours in adolescents with comorbid LD-ADHD (r = 0.57) [24]. Regarding externalizing behaviours, Al-Yagon et al. found a significant positive correlation with loneliness (peer network (r = 0.28) and peer-dyadic (r = 0.33)) in adolescents with LD [24].

Several studies focused on child socio-emotional development, specifically on parental attachment, affects, social skills development, and family functioning. Adolescent-parent attachment was studied by Al-Yagon et al. in 2016 among adolescents with LD or comorbid LD with ADHD [24]. In both populations, they found a statistically significant negative correlation between peer network loneliness and adolescent–mother attachments or father attachments (see Table 1) [24]. In the same populations, Al-Yagon et al. also found both a significant positive correlation between loneliness and negative affect and a negative correlation between loneliness and positive affect (Table 1) [24]. The association between loneliness and social skills was studied by Zach, Yazdi-Ugav, and Zeev (2016). This group found that students with LD had lower scores in social skills [32]. They also uncovered that learning disorders and loneliness did not contribute to social skills variance in girls, although loneliness could explain part of the variance in boys [32]. Finally, in adolescents with LD, Idan and Margalit (2014) found a statistically significant negative correlation between loneliness and family cohesion (Spearman correlation of −0.18, *p* < 0.01) [35]. Family cohesion in this study was measured with a subscale of the Family Adaptability and Cohesion Evaluation Scale and referred to emotional bonding, family boundaries, and time spent together.

The association between loneliness and academics outcomes was explored in two studies. In youth with LD, Lackaye & Margalit (2008) showed that loneliness was negatively correlated with academic self-efficacy (Pearson correlation of −0.26) [34]. Meanwhile, Idan and Margalit (2014) found in the same population a statistically positive correlation between loneliness and English self-efficacy (Spearman correlation of 0.25) [35].

A couple of studies investigated the link between loneliness and hope in adolescents with LD. They both found a statistically significant negative correlation between loneliness and hope: Spearman correlation of −0.42 from Idan and Margalit (2014) and Pearson correlation of −0.42 from Lackaye & Margalit (2008) [34,35]. In 1999, Valas identified a significant negative correlation between loneliness and self-esteem in youth with LD (Pearson correlation of −0.251) [33]. Similarly, in 2013, Tillinger uncovered that age 10 loneliness could significantly predict global self-worth at age 18 in those with developmental disabilities; further, higher loneliness at age 10 was related to lower levels of self-worth at age 18 [43].

Finally, a sense of coherence defined as “a global orientation that expresses the extent to which one has a pervasive, enduring feeling of confidence that the stimuli deriving from one’s environment are structured, predictable, and explicable” was studied by Idan and Margalit (2014) [35]. They found in adolescents with LD a statistically significant negative correlation between loneliness and sense of coherence (Spearman correlation of −0.43) [35]. Idan and Margalit (2014) also detected a significant negative correlation between loneliness and basic psychological needs such as autonomy, competence, and relatedness [35]. The correlation coefficients can be found in Table 1.

### 3.6. Quality Appraisal

A quality assessment of the included studies was done using the QATSDD tool and can be found in Table 2. The included studies scored between 19 to 38 out of a maximum score of 42 for either quantitative or qualitative studies, or a total maximum score of 46 for mixed-methods studies. None of the studies scored below 15. Six studies scored between 15–28 and six studies scored above 30.

## 4. Discussion

To our knowledge, this is the first review of the literature that focuses on the association between loneliness/social isolation and health/developmental problems in children with NDD. Our review led to the inclusion of 12 articles and although most articles originated from the USA, there were articles from three other countries (Israel, Australia, and Norway), suggesting that loneliness/social isolation in children with NDD is a worldwide issue.

One interesting result is the preference for studying the effects of loneliness in 10 out of 12 studies instead of social isolation, investigated in 3 out of 12 studies. Although isolation can lead to loneliness [50], loneliness better reflects the psychosocial and emotional consequences of “not feeling adequately included in supportive groups and relationships.” [51]. Similarly, Heinrich and Gullone’s review on loneliness reports “the absence of a needed relationship” as one of the main causes of distress due to loneliness [8]. Considering Vygotsky’s model of socio-cultural learning [25], it seems that both isolation and loneliness may affect the child’s learning and development; however, when this leads to loneliness, this can have additional consequences, mainly on mental health as well as behavioural and socio-emotional development. It would be interesting in future research to investigate separately the specific impact and relationships of social isolation and loneliness as these two different concepts may deserve different interventions.

Overall this review revealed that in children with NDD, loneliness was associated with mental health problems, developmental challenges (behavioural and socio-emotional), and learning limitations. Loneliness in children with NDD was positively correlated with depressive symptoms and anxiety and negatively correlated with many other outcomes, such as hope, sense of coherence, basic psychological needs, and self-esteem. These factors are all inter-related and certainly contribute to depression. These findings in children with NDD are consistent with the literature on loneliness in both adults with NDD and in the general pediatric population [52,53,54,55,56,57,58]. For instance, in a cross-sectional study with 108 adults with ASD, Mazurek showed that loneliness was positively correlated with depression (r = 0.48, *p* < 0.001) and anxiety (r = 0.34, *p* = 0.001) [52]. Likewise, Loades et al. conducted a rapid review in 2020 to investigate the impact of loneliness on the mental health of children and adolescents that were previously healthy [55]. Their review included 63 studies in their synthesis and they found a clear association between loneliness and mental health problems (depression and anxiety) in the pediatric population [55]. In short, like other populations, children with NDD suffer when they are lonely.

Another main finding from our review was the long-lasting impact of loneliness in childhood, which could extend to adulthood. This is consistent with the literature, where a lasting impact of loneliness into adulthood has also been found in the general child population. In a 2006 longitudinal study done by Caspi et al. that followed 1037 children from birth to age 26, it was shown that compared with non-isolated children, socially isolated children were at significant risk of poor adult health; a 1 SD change in childhood social isolation increased the risk of adult risk factor clustering (defined as having adverse levels of 3 or more of the 6 adult biomarkers) by 1.37. This association was found to be independent of other childhood risk factors for poor adult health, such as low childhood socioeconomic status, low childhood IQ, and being overweight in childhood. This association was also not accounted for by behaviours that were health damaging and it was not attributable to life events that were stressful [12]. Similarly, Loades et al.’s rapid review found that loneliness was associated with future mental health problems up to 9 years later [55]. The longitudinal study of loneliness across the lifespan is certainly extremely important to better ascertain the complex role of certain factors that can be both contributors or consequences of loneliness, but is critically missing from the literature.

We also found that loneliness was negatively correlated with academic self-efficacy, which is consistent with literature on the general pediatric population [35,59]. However, loneliness was also found in our scoping review in one study to be positively correlated with English self-efficacy, which seems to be the only outcome positively associated with loneliness [35]. Overall, the impact of loneliness on academic achievement is unclear [59,60], probably in relation to the primary disability that may affect the intellectual capacity of the child. For instance, children with Asperger syndrome, while having social communication deficits, may also have excellent academic achievements. Griswold et al.’s study assessed academic achievement in 21 children with Asperger syndrome using the Wechsler Individual Achievement Test (WIAT) and found that the scores varied greatly from significantly below to significantly above average [61]. Similarly, children with DS also have a range of academic attainment [62] and their academic attainment can be affected by various factors such as attendance at a mainstream school and maternal coping style [63]. The relationship between loneliness and academic performance can, therefore, vary depending on the NDD itself as well as environmental factors.

Our review also reveals that currently in the literature, the associations with loneliness have only been studied mainly in the following NDD diagnoses: ASD, LD, and ADHD. In youth with ASD, our review found that loneliness has been associated with depression, suicidal ideation, and anxiety. Interestingly, these studies were all conducted in the older pediatric population, mainly including teenagers rather than children. This may be because loneliness is more prevalent in the adolescent group in general. Another reason may be because as children with ASD get older, they may start to find other people interesting and want to make friends [20]. It would be interesting to see if younger children with ASD experience the same mental health associations with loneliness. In terms of internalizing behaviours, there were mixed findings in the ASD population. One study found no correlation between number of friends and internalizing behaviours, whereas another found a negative correlation between social participation and internalizing behaviours. This mixed finding might be explained by the different degrees of ASD severity in the study participants; for instance, one of the two studies included children who were non-verbal, so the parents filled out the questionnaires on their behalf. Hence, there may be bias in the results as the internalizing behaviours reflect the parents’ interpretation and not necessarily the child’s perception. This mixed finding might also be because the mood of those with ASD is not overly modulated by ostracism, even if data suggest that they are able to recognize when they are being excluded [64]. Peristeri et al. conducted a study involving 21 participants with high-functioning autism and found that they failed to interpret their emotional state appropriately [65]. Even if they may feel as excluded as their controls, they might lack insight into how that affected their mood [65].

In the LD population (with or without comorbid ADHD), the association between loneliness and both internalizing and externalizing behaviours clearly showed a positive correlation between loneliness and these behaviours. This clearer finding compared to the ASD population may reflect the more significant social communication deficits in those with ASD and the difficulty in interpreting their emotional state. In adolescents with LD, loneliness has also been negatively associated with hope and positively associated with depression. Again, like in the ASD population, it would be interesting to see if younger children with LD experience the same mental health associations with loneliness.

In children with ADHD only, loneliness was found to be associated with anxiety. Interestingly, the effect size was larger in children with ADHD compared to youth with ASD (beta of 0.62 vs. 0.24) when looking at the relationship between loneliness and mental health problems (depression or anxiety). This may be due to challenges with diagnosing depression in individuals with ASD. With their communication deficits, individuals with ASD may not be able to directly express feelings of sadness, guilt, or low self-esteem [66]. Their clinical presentation of depression may also be more atypical and more difficult to recognize. For instance, their affect is often flat or restricted, so changes associated with depression may be more difficult to recognize [66].

Our scoping review identified several gaps in the areas of study design, study participants, and study outcomes. The majority of the studies on loneliness (90%) used a cross-sectional design. Cross sectional assessment of loneliness and outcomes is strongly limited in assessing the direction of the associations, which makes it difficult to identify risk factors; factors can be either a cause or consequence of loneliness. For instance, this review noticed the bidirectional relationship between loneliness and depression, where loneliness may lead to depression but increased depression may also lead to decreased self-esteem, which may contribute to increased social retraction and higher feelings of loneliness. The only prospective study by Tillinger [43] is by far the most informative, but it also has possible biases that limit causal inference. Hence, firm conclusions on the effects of loneliness on children with NDD cannot be drawn from this review.

Another gap in this area is that many studies had a relatively small sample size, which would also make it difficult to draw firm conclusions or generalize the results. Moreover, only several NDD diagnoses have been studied, such as ASD, LD, ADHD, and Down syndrome. Some of these diagnoses were only studied once or twice, including ADHD and Down syndrome. Many diagnoses would be worth studying in the future, such as developmental coordination disorder, fetal alcohol syndrome disorder (FASD), or tic disorder.

Furthermore, although mental health outcomes and certain child developmental domains outcomes (behavioural, socioemotional) were investigated, physical health outcomes and cognitive domain of child development were not. It is well known in the adult literature that loneliness is associated with poor physical health outcomes, such as heart disease, hypertension, stroke, lung disease, and metabolic disorders [11]. It would thus be of interest to assess physical activity level and physical health outcomes in children with NDD as well as their relation to loneliness; this can potentially identify an area that deserves early intervention and prevent long term health consequences from developing in adulthood. Motor function is also important for child learning and development and would be worth investigating. Social skills and academics are also important areas in children’s development, but they were included in only three articles. Overall, there was more of a focus on the impact of mental health than learning and development. More research in these aforementioned areas would be beneficial and on the relationship between these areas and social isolation, with a special focus on bidirectional relationships that may create negative reinforcement loops.

Given all the potential negative effects of loneliness on children with NDD and the long term consequences, we expected to find more research in this area and more high quality research with prospective follow up to understand the mechanism of loneliness development and the impact on child development and health, especially on mental health. One may wonder if the lack of research is because loneliness is not yet considered a serious issue.

Besides the special care and treatment for children with NDD, it is important to consider specific interventions to decrease isolation and loneliness, which could improve some aspects of the child’s mental wellness and development. For instance, Matthews et al. conducted a randomized control trial in a sample of 34 adolescents with ASD, comparing the traditional PEERS curriculum (14-week intervention that teaches social and friendship skills to teens with ASD via didactic lessons, role plays, and behavioural rehearsals) to a peer-mediated PEERS curriculum and delayed treatment control group [67]. This trial revealed that there was an advantage in social skills and social functioning for those in the peer-mediated PEERS curriculum compared to the traditional PEERS curriculum, and the improvements in social skills, social functioning, and loneliness were maintained at the 4 month follow up [68]. Likewise, Elmose and Lasgaard found in a sample of 224 adolescents (25 with ADHD, 199 without) that social support from peers can decrease loneliness in the ADHD group [69]. Hence, there is evidence that interventions such as peer support can help decrease loneliness in children with NDD, which can help circumvent the potential consequences of loneliness, independent of the primary disability.

Like all scoping reviews, ours too has limitations because the focus is on breadth rather than depth. However, this methodological design is appropriate since this is the first review in the area of loneliness in children with NDD, so our research question is broad and our goal is to map out the evidence and identify any gaps in the literature. Another limitation is the restriction of included studies based on the language; our results may thus only be generalizable to articles written in English or French.

In summary, this scoping review revealed that in children with NDD, there are potential consequences of loneliness on their mental health (depression and anxiety), learning, and development (both socio-emotional and behavioural). These effects could also be lasting, extending into adulthood. In the future, it would be beneficial to conduct a systematic review on loneliness in children with NDD and more specific areas (e.g., depression or child cognitive functions development), especially when more articles are published in this field of research. New studies should adopt longitudinal designs with larger sample sizes and which involve various NDD diagnoses. More of a focus around the impact on learning, development (especially cognitive), and physical health would also be valuable. These prospective studies should focus on understanding the model “from exclusion to loneliness” and assessing the dynamic changes that demonstrate how an excluded child can become lonely, with long-term consequences into adulthood. Finally, there is a need to develop and evaluate interventions that promote social inclusion/participation to change the pattern of loneliness/social isolation with the goal of circumventing their potential consequences. Besides the positive effects they may have on children, intervention studies are important to understand the causal relationship between loneliness and depression or other diseases. In contrast with the positive effects of interventions aimed at breaking loneliness, the lack of research in this area suggests that loneliness is not yet considered an independent serious issue in children with NDD that deserves specific attention and the development of possible interventions.

## Figures and Tables

**Table 1:** Summary of included studies.

Study	Country	Design	Sample Size (N, Female)	Age Range (Mean)	Demographics	Diagnosis	Outcomes Measured	Measurement Tools	Key Findings
Zach, Yazdi-Ugav, Zeev. (2016) [32]	Israel	Cross-sectional	733 (374 females)	6.04–13.72 years (8.82)	General education class in 2 Public schools, grade 1–5 in Israel. LD: +2 SD below average achievement in standardized tests and IQ 85–115	LD, TD	1. Social skills 2. Social dissatisfaction	1. Elementary school Social Skills Rating System (SSRS)-T form - Hebrew Adaptation, completed by teacher and includes 3 domains: social skills, problem behaviours, and academic competence. Loneliness was measured with a modified version of the Loneliness and Social Dissatisfaction questionnaire by Asher.	- Learning disorders and loneliness did not contribute to the explanation of the females’ social skills variance. In boys, loneliness can explain their social skills variance.
ValÅs (1999) [33]	Norway	Cross-sectional	1434 (728 females)	10–16 years (not reported)	552 4th grade, 511 7th grade, 371 9th grade from 128 classes across 49 schools in Norway in 1996.	LD, low-achieving	1. Peer acceptance 2. Self-esteem Depression	1. Sociometric question referred to the hypothetical situation: “Imagine the school will form new classes and you can decide who goes into which classes. Which students in your grade would you like to have in your new class?” 2. A scale modified from the Self-Description Questionnaire SDQ II 3. Scale modelled after Kovacs’ 1985 instrument that assesses affective, cognitive, motivational, and somatic symptoms of depression. Loneliness was measured with the Loneliness and Social Dissatisfaction Questionnaire by Asher (modified version).	- Pearson correlations were significant between loneliness and the following: self-esteem (r = −0.251), depression (r = 0.312) in all participants. Students with LD compared with non-LD and non low-achieving students were less accepted among peers, were more lonely, and had lower self-esteem.
Lackaye & Margalit (2008) [34]	Israel	Cross-sectional	280 (140 females)	2 age groups: 7th grade and 10th grade	From 10 schools in Israel. LD students: IQ 85–120	LD and non-LD	1. Numerical grades for mathematics and history 2. Specific academic self-efficacy 3. General academic self-efficacy 4. Hope 5. Effort	1. Grade reports 2. The Hebrew adaptation of the Specific Academic Self-Efficacy Scale 3. The Hebrew adaptation of the General Academic Self-Efficacy Scale 4. The Hebrew adaptation of The Children’s Hope Scale 5. Adaptation of the Meltzer scale for effort Loneliness was measured with the Hebrew adaptation of the Loneliness and Social Dissatisfaction Questionnaire from Asher	Loneliness is statistically significantly correlated with: academic self-efficacy (r = −0.35), history (r = 0.20), and hope (r = −0.42).
Idan & Margalit (2014) [35]	Israel	Cross-sectional	856 (396 females)	15–18 years (not reported)	Public High school students from seven high schools in Central Israel (5 located in predominantly middle class districts, 2 located in predominantly lower class districts) LD students: IQ 85–120, achievement scores at least one standard deviation	LD and non-LD	1. Sense of coherence 2. Basic psychological needs (autonomy, competence, relatedness) 3. Family climate (cohesiveness, adaptability) 4. Hope 5. Effort 6. Academic self efficacy Students’ achievements	1. Sense of coherence scale 2. Basic psychological needs scale 3. FACES III (family adaptability and cohesion evaluation scale) 4. Hebrew adaptation of the children’s hope scale 5. Effort scale 6. Hebrew adaptation of the academic self-efficacy scale 7. Students’ numerical grades Loneliness was measured with the Hebrew adaptation of the Loneliness Scale from Asher.	- In the LD group, there was a statistically significant negative correlation between loneliness and the following measures (spearman correlation in bracket): sense of coherence (−0.43), autonomy/competence (−0.42), relatedness (−0.64), family cohesion (−0.18). A positive correlation with self-efficacy English (0.25), Loneliness contributed negatively to hope (−0.42).
Al-Yagon (2016) [24]	Israel	3 groups Cross-sectional assessment	280 (154 females)	15–17 years (15.94)	Grade 10/11 public high schools in urban Israel. Parents: mainly married full time workers with a university degree.	LD, LD + ADHD, TD	1. Adolescents’ (ado) perceptions of security in parent-child relationship. 2. Ados’ perceptions of their homeroom teacher as an attachment figure. 3. Ados’ perception of their relationship with their very best friend. 4. Ados’ view of their own affects. 5. Emotional and behavioural problems among youth	1. Attachment security style scale (answered by teen) 2. Children’s Appraisal of Teacher as a Secure Base Scale. 3. Friendship Quality Questionnaire. 4. Affect scale. 5. Externalizing/Internalizing Syndrome scales from the standardized Youth Self-Report Version for Age 11–18 Loneliness was measured with (i) the Peer-Network Loneliness and (ii) the Peer-Dyadic Loneliness Scale.	Ado with comorbid LD + ADHD: - significant correlation between peer network loneliness and: ado-mother attachment (r = −0.31), ado-father attachment (r = −0.27), teacher’s availability (r = −0.21), teacher’s rejection (r = 0.25), friendship quality (r = −0.47), positive affect (r = −0.47), negative affect (r = 0.39), internalizing behaviours (r = 0.57). - significant correlation between peer-dyadic loneliness and: adolescent mother attachment (r = −0.22), friendship quality (−0.55), positive affect (r = −0.30). Ado with LD only: - significant correlation between peer network loneliness and: ado-mother attachment (r = −0.38), ado-father attachment (r = −0.25), friendship quality (−0.41), positive affect (r = −0.4), negative affect (r = 0.35), externalizing behaviours (r = 0.28), internalizing behaviours (r = 0.45). - significant correlation between peer dyadic loneliness and: ado mother attachment (r = −0.25), friendship quality (r = −0.49), positive affect (r = −0.37), negative affect (r = 0.27), externalizing behaviour (r = 0.33), internalizing behaviour (r = 0.47)
Becker (2015) [36]	United States	Cross-sectional	112 (39 females)	7–12 years (8.79)	76% non-Hispanic white, 17% African American, 5% Hispanic, 2% Asian, 1% Native American. 9% annual family income >$20,000, 34% $20,001–50,000, 21% $50,001–80,000, 36% <$80,000	ADHD-I or ADHD-C	1. Academic achievement 2. Intelligence 3. Social Information Processing attribution biases (internal and external attributions) 4. oppositional defiant/conduct disorder (ODD/CD) symptoms 5. Anxiety symptoms 6. Positive illusory bias 7. Aggression 8. Perceived social acceptance 9. Peer isolation	1. Wechsler Individual Achievement Test 2. The Kaufman Brief Intelligence Test Second Edition 3. Externalizing-relevant vignettes (responses coded as negative internal, negative external, or neutral attributions) and internalizing-relevant vignettes (Children’s Evaluation of Everyday Social Encounters Questionnaire) 4. The Kiddie Schedule for Affective Disorders and Schizophrenia for School-Age Children, Vanderbilt ADHD Diagnostic Rating Scale 5. The Revised Child Anxiety & Depression Scales 6. Self-Perception Profile for Children (SPPC) 7. Dodge and Coie’s (1987) measure of aggressive behaviors 8. The Child Behavior Scale Loneliness was measured with the Loneliness Questionnaire (Asher et al., 1984) [37].	- Peer exclusion/withdrawal not significantly correlated with child depressive symptoms or anxiety symptoms. - ODD/CD symptoms were significantly associated with peer exclusion (r = 0.26, *p* = 0.005). - Negative internal attribution bias had a significant negative association with peer withdrawal - Anxiety was negatively associated with perceived social acceptance (β = −0.38, SE = 0.09, *p* < 0.001) and positively associated with loneliness (β = 0.62, SE = 0.06, *p* < 0.001).
Dovgan & Mazurek (2019) [38]	United States	Cross-sectional, survey	129 (18 females)	6–18 years (10.86)	40.3% scored IQ less than 70	ASD	1. Adaptive Behaviour 2. Childhood emotional & behavioural problems 3. Internalizing & externalizing symptoms 4. Participation in sports, hobbies and clubs, total activities 5. Friendships 6. Intelligence (IQ)	1. Vineland Adaptive Behaviour Scales 2. The Child Behavior Checklist. 3. Early Years Differential Ability Scales, School-Age Differential Ability Scales, Wechsler Abbreviated Scale of Intelligence or Mullen Scales of Early Learning Social participation/isolation was measured by the Child Behaviour Checklist.	- Internalizing problems were not significantly correlated with the number of friends (rs = 0.010, *p* = 0.914) or with the number of total activities (r = 0.037, *p* = 0.678).
White & Roberson-nay (2009) [39]	United States	Cross-sectional	20 (2 females)	7–14 years (12.08)	95% attended public regular education schools. 14 received special education services, 8 received speech/language therapy, 5 received social skills training interventions. 65% on medication	Clinical diagnoses of ASD including autistic disorder, PDD-NOS, or AS	1. Prosocial index and social initiative index 2. Anxiety 3. Social competence, emotional and behavioral problems	1. Social Communication Questionnaire 2. Social Responsiveness Scale 3. Social Competence Inventory 4. Multidimensional Anxiety Scale for Children 5. Child Behavior Checklist Loneliness was measured with the Loneliness Questionnaire by Asher.	- The high-anxiety group self-reported more social loneliness than their less anxious peers (t = 2.57, *p* < 0.05). - Social and global loneliness scores were significantly correlated with social anxiety (r = 0.59, *p* = 0.01) and (r = 0.50, *p* = 0.04), respectively.
Whitehouse et al. (2009) [40]	Australia	Cross-sectional	70 (13 females)	AS group: 12–17 years (14, 2 mos) TD group: 13–16 years (14, 4 mos)	Attending mainstream secondary schools in one of three states in Australia: Western Australia, New South Wales, and Queensland.	Asperger’s syndrome, TD	1. Friendship Depressive symptom	1. Friendship Quality Questionnaire and Friendship Motivation Questionnaire 2. Centre for Epidemiological Studies Depression Scale Children’s Version Loneliness was measured with the De Jong-Gierveld Loneliness Scale.	- Loneliness significantly mediated the association between the conflict/betrayal subscale (*p* < 0.01) and levels of depressive symptoms in the AS group (*p* < 0.001), which was the only significant predictor of depression.
Lounds Taylor, Adams, Bishop (2017) [41]	United States	Longitudinal	36 (6 females)	17–22 years (18.71)	88.9% white non-Hispanic IQ range 40–147 standard score Avg 85.33 (SD25.65) Schooling: 63.9% regular public/magnet school, 8.3% regular private school, 11.1% school that only serves students with disabilities, 13.9% home schooled, 2.8% other All youth living with parents Parents: 32 mothers and 4 fathers, age 38–59. 69.4% married, 69.4% post- secondary degree, 30.6% post-bachelor’s degree Avg annual income $85,000, 25% <$50,000	ASD	Internalizing symptoms	1. Adult behaviour checklist Social Participation was measured by the National Survey of Families and Households, modified to be appropriate for adolescents and adults	- The correlation between earlier unstructured social participation and later internalizing symptoms was −0.26.
Hedley et al. (2018) [42]	Australia	Cross-sectional, survey	185 (92 females)	14–80 years (37.11)	Employment: 49.7% employed (part-time/full-time). Education: 7.2% Current secondary, 3.8% Some secondary, 9.2% completed secondary, 21.1% certificate or diploma, 27.6% Bachelor’s degree, 18.9% Post graduate degree, 11.9% other/not reported. Living Conditions: 31.9% w/parents, 2.2% relatives, 6.5% Others, 20.5% alone, 34.1% couples, 4.9% other	ASD	1. Social support 2. Major and subthreshold depressive disorder and suicidal ideation	1. Social Support Questionnaire-Shortened Version (SSQ-6) 2. Patient Health Questionnaire (PHQ) Loneliness was measured with the University of California Los Angeles Loneliness Scale-Short Form	- Loneliness was negatively correlated with social support satisfaction/number (𝛽 = –0.47, *p* < 0.001), but positively correlated with depression (𝛽 = 0.24, *p* = 0.002) and suicidal ideation (b = 0.04; BCa [0.02, 0.06]). Depression predicted suicidal ideation(𝛽 = 0.51, *p* < 0.001).
Tillinger (2013) [43]	United States	Longitudinal data, correlational design	93 (49 females)	Recruited at less than 24 months old, then home visits were conducted when children were age 1, 2, 3, 5, 10, 18, and 23 years.	90% Euro-American descent from Massachusetts and New Hampshire. Family avg annual $40,000–45,000. Mothers’ education = avg 13.97 yrs. Child IQ 62.4% lower than one standard deviation below standardized mean, 53.8% lower than two standard deviations below standardized mean.	Down syndrome, motor impairment, or developmental delay of unknown etiology	1. Child cognitive functioning 2. Autonomy 3. Self-efficacy 4. Behaviour problems 5. Nature of friend relationships at age 18 6. Friendship quality 7. Family satisfaction 8. Adolescent global self-worth 9. Adolescent depressive symptoms	1. Stanford-Binet Intelligence Scales 2. Arc’s Self Determination Scale–Autonomy Subscale 3. Perceived Self-Efficacy Scale 4. Child Behavior Checklist 5. Multidimensional Scale of Perceived Social Support–Friends Subscale 6. Pictorial Scale of Perceived Competence and Social Acceptance for Young Children 7. Maternal Acceptance Subscale 8. Self-Perception Profile for Learning Disabled Students-Global Self-worth Subscale 9. Center for Epidemiologic Studies Depression Scale Loneliness was measured with the Loneliness and Social Dissatisfaction Questionnaire (Williams & Asher, 1992).	- Age 10 loneliness did not significantly predict friendship quality at age 18. - Age 10 loneliness was found to significantly predict adolescent global self-worth at age 18 (*p* = 0.008); higher levels of loneliness at age 10 led to lower levels of adolescent global self-worth at age 18. - Age 10 loneliness was found to significantly predict adolescent depressive symptoms at age 18 (*p* = 0.013); higher levels of loneliness at age 10 led to higher levels of adolescent depressive symptoms at age 18.

Acronyms: ADHD = attention deficit/hyperactivity disorder, (predominantly inattentive (ADHD-I), combined inattentive/hyperactive-impulsive (ADHD-C)), AS = Asperger’s Syndrome, ASD = Autism Spectrum Disorder, LD = learning disabilities, PDD-NOS = Pervasive Developmental Disorder–Not Otherwise Specified, TD = typical development.

**Table 2:** Quality Assessment of the included studies based on the QATSDD tool.

Criteria	Al-Yagon [24]	Becker [36]	Dovgan & Mazurek [38]	Hedley et al. [42]	Idan & Margalit [35]	Lackaye & Margalit [34]	Lounds Taylor, Adams, Bishop [41]	Tillinger [43]	Valas [33]	White & Robertson-nay [39]	Whitehouse et al. [40]	Zach, Yazdi-Ugav, Zeey [32]
Explicit theoretical framework	3	3	3	3	3	2	2	3	3	3	2	2
Statement of aims/objectives in the main body of the report	3	3	3	3	3	3	3	3	3	3	3	2
Clear description of research setting	3	3	3	2	3	3	2	3	3	2	3	3
Evidence of sample size considered in terms of analysis	0	1	0	0	0	0	1	3	0	0	0	0
Representative sample of target group of a reasonable size	2	2	1	3	3	2	1	2	3	1	1	2
Description of procedure for data collection	3	3	3	3	3	3	3	3	3	2	2	1
Rationale for choice of data collection tool(s)	2	2	2	3	3	2	2	3	3	2	1	1
Detailed recruitment data	3	3	1	3	1	2	2	1	2	2	2	1
Statistical assessment of reliability and validity of measurement tool(s) (Quantitative only)	1	2	1	3	2	2	1	2	2	1	2	2
Fit between stated research question and method of data collection (Quantitative)	2	2	2	3	2	1	1	2	2	2	2	1
Fit between stated research question and format and content of data collection tool e.g., interview schedule (Qualitative)	n/a	2	2	3	3	1	1	1	2	2	2	1
Fit between research question and method of analysis	3	3	2	3	2	1	2	3	2	1	1	1
Good justification for analytical method selected	3	2	2	3	3	2	1	2	2	2	1	1
Assessment of reliability of analytical process (Qualitative only)	n/a	3	0	0	1	0	0	0	0	0	0	0
Evidence of user involvement in design	0	0	0	0	0	0	0	0	0	0	0	0
Strengths and limitations critically discussed	3	2	3	3	1	2	2	2	1	1	1	1
**Total score**	31	36	28	38	33	26	24	33	31	24	23	19

n/a: not applicable.

## Appendix A. Complete Search Strategy for Each Database

MEDLINE:

1. exp Neurodevelopmental Disorders/

2. neurodevelopmental dis*.mp. [mp = title, abstract, original title, name of substance word, subject heading word, floating sub-heading word, keyword heading word, organism supplementary concept word, protocol supplementary concept word, rare disease supplementary concept word, unique identifier, synonyms]

3. Anxiety.mp. [mp = title, abstract, original title, name of substance word, subject heading word, floating sub-heading word, keyword heading word, organism supplementary concept word, protocol supplementary concept word, rare disease supplementary concept word, unique identifier, synonyms]

4. Attention Deficit.mp. [mp = title, abstract, original title, name of substance word, subject heading word, floating sub-heading word, keyword heading word, organism supplementary concept word, protocol supplementary concept word, rare disease supplementary concept word, unique identifier, synonyms]

5. Child Behavior Disorder.mp. [mp = title, abstract, original title, name of substance word, subject heading word, floating sub-heading word, keyword heading word, organism supplementary concept word, protocol supplementary concept word, rare disease supplementary concept word, unique identifier, synonyms]

6. conduct disorder.mp. [mp = title, abstract, original title, name of substance word, subject heading word, floating sub-heading word, keyword heading word, organism supplementary concept word, protocol supplementary concept word, rare disease supplementary concept word, unique identifier, synonyms]

7. Child Development Disorder.mp. [mp = title, abstract, original title, name of substance word, subject heading word, floating sub-heading word, keyword heading word, organism supplementary concept word, protocol supplementary concept word, rare disease supplementary concept word, unique identifier, synonyms]

8. autism.mp. [mp = title, abstract, original title, name of substance word, subject heading word, floating sub-heading word, keyword heading word, organism supplementary concept word, protocol supplementary concept word, rare disease supplementary concept word, unique identifier, synonyms]

9. Communication Disorder.mp. [mp = title, abstract, original title, name of substance word, subject heading word, floating sub-heading word, keyword heading word, organism supplementary concept word, protocol supplementary concept word, rare disease supplementary concept word, unique identifier, synonyms]

10. developmental dis*.mp. [mp = title, abstract, original title, name of substance word, subject heading word, floating sub-heading word, keyword heading word, organism supplementary concept word, protocol supplementary concept word, rare disease supplementary concept word, unique identifier, synonyms]

11. intellectual dis*.mp. [mp = title, abstract, original title, name of substance word, subject heading word, floating sub-heading word, keyword heading word, organism supplementary concept word, protocol supplementary concept word, rare disease supplementary concept word, unique identifier, synonyms]

12. learning dis*.mp. [mp = title, abstract, original title, name of substance word, subject heading word, floating sub-heading word, keyword heading word, organism supplementary concept word, protocol supplementary concept word, rare disease supplementary concept word, unique identifier, synonyms]

13. motor skills dis*.mp. [mp = title, abstract, original title, name of substance word, subject heading word, floating sub-heading word, keyword heading word, organism supplementary concept word, protocol supplementary concept word, rare disease supplementary concept word, unique identifier, synonyms]

14. tic dis*.mp. [mp = title, abstract, original title, name of substance word, subject heading word, floating sub-heading word, keyword heading word, organism supplementary concept word, protocol supplementary concept word, rare disease supplementary concept word, unique identifier, synonyms]

15. mutism.mp. [mp = title, abstract, original title, name of substance word, subject heading word, floating sub-heading word, keyword heading word, organism supplementary concept word, protocol supplementary concept word, rare disease supplementary concept word, unique identifier, synonyms]

16. schizophrenia.mp. [mp = title, abstract, original title, name of substance word, subject heading word, floating sub-heading word, keyword heading word, organism supplementary concept word, protocol supplementary concept word, rare disease supplementary concept word, unique identifier, synonyms]

17. movement dis*.mp. [mp = title, abstract, original title, name of substance word, subject heading word, floating sub-heading word, keyword heading word, organism supplementary concept word, protocol supplementary concept word, rare disease supplementary concept word, unique identifier, synonyms]

18. reactive attachment dis*.mp. [mp = title, abstract, original title, name of substance word, subject heading word, floating sub-heading word, keyword heading word, organism supplementary concept word, protocol supplementary concept word, rare disease supplementary concept word, unique identifier, synonyms]

19. (Down syndrome or trisomy 21).mp. [mp = title, abstract, original title, name of substance word, subject heading word, floating sub-heading word, keyword heading word, organism supplementary concept word, protocol supplementary concept word, rare disease supplementary concept word, unique identifier, synonyms]

20. exp Down Syndrome/

21. exp Cerebral Palsy/

22. cerebral palsy.mp. [mp = title, abstract, original title, name of substance word, subject heading word, floating sub-heading word, keyword heading word, organism supplementary concept word, protocol supplementary concept word, rare disease supplementary concept word, unique identifier, synonyms]

23. 1 or 2 or 3 or 4 or 5 or 6 or 7 or 8 or 9 or 10 or 11 or 12 or 13 or 14 or 15 or 16 or 17 or 18 or 19 or 20 or 21 or 22

24. exp Loneliness/

25. lonel*.mp. [mp = title, abstract, original title, name of substance word, subject heading word, floating sub-heading word, keyword heading word, organism supplementary concept word, protocol supplementary concept word, rare disease supplementary concept word, unique identifier, synonyms]

26. exp Social Isolation/

27. social* isolat*.mp. [mp = title, abstract, original title, name of substance word, subject heading word, floating sub-heading word, keyword heading word, organism supplementary concept word, protocol supplementary concept word, rare disease supplementary concept word, unique identifier, synonyms]

28. exp Social Participation/

29. social* participat*.mp. [mp = title, abstract, original title, name of substance word, subject heading word, floating sub-heading word, keyword heading word, organism supplementary concept word, protocol supplementary concept word, rare disease supplementary concept word, unique identifier, synonyms]

30. 24 or 25 or 26 or 27 or 28 or 29

31. 23 and 30

32. limit 31 to “all child (0 to 18 years)”

EMBASE:

1. exp mental disease/or exp autism/or exp behavior disorder/or exp learning disorder/

2. neurodevelopmental dis*.mp. [mp = title, abstract, original title, name of substance word, subject heading word, floating sub-heading word, keyword heading word, organism supplementary concept word, protocol supplementary concept word, rare disease supplementary concept word, unique identifier, synonyms]

3. anxiety.mp. [mp = title, abstract, original title, name of substance word, subject heading word, floating sub-heading word, keyword heading word, organism supplementary concept word, protocol supplementary concept word, rare disease supplementary concept word, unique identifier, synonyms]

4. Attention Deficit.mp. [mp = title, abstract, original title, name of substance word, subject heading word, floating sub-heading word, keyword heading word, organism supplementary concept word, protocol supplementary concept word, rare disease supplementary concept word, unique identifier, synonyms]

5. Child Behavio* Dis*.mp. [mp = title, abstract, original title, name of substance word, subject heading word, floating sub-heading word, keyword heading word, organism supplementary concept word, protocol supplementary concept word, rare disease supplementary concept word, unique identifier, synonyms]

6. Child Development Dis*.mp. [mp = title, abstract, original title, name of substance word, subject heading word, floating sub-heading word, keyword heading word, organism supplementary concept word, protocol supplementary concept word, rare disease supplementary concept word, unique identifier, synonyms]

7. autism.mp. [mp = title, abstract, original title, name of substance word, subject heading word, floating sub-heading word, keyword heading word, organism supplementary concept word, protocol supplementary concept word, rare disease supplementary concept word, unique identifier, synonyms]

8. communication dis*.mp. [mp = title, abstract, original title, name of substance word, subject heading word, floating sub-heading word, keyword heading word, organism supplementary concept word, protocol supplementary concept word, rare disease supplementary concept word, unique identifier, synonyms]

9. developmental dis*.mp. [mp = title, abstract, original title, name of substance word, subject heading word, floating sub-heading word, keyword heading word, organism supplementary concept word, protocol supplementary concept word, rare disease supplementary concept word, unique identifier, synonyms]

10. intellectual dis*.mp. [mp = title, abstract, original title, name of substance word, subject heading word, floating sub-heading word, keyword heading word, organism supplementary concept word, protocol supplementary concept word, rare disease supplementary concept word, unique identifier, synonyms]

11. learning dis*.mp. [mp = title, abstract, original title, name of substance word, subject heading word, floating sub-heading word, keyword heading word, organism supplementary concept word, protocol supplementary concept word, rare disease supplementary concept word, unique identifier, synonyms]

12. motor skills dis*.mp. [mp = title, abstract, original title, name of substance word, subject heading word, floating sub-heading word, keyword heading word, organism supplementary concept word, protocol supplementary concept word, rare disease supplementary concept word, unique identifier, synonyms]

13. tic dis*.mp. [mp = title, abstract, original title, name of substance word, subject heading word, floating sub-heading word, keyword heading word, organism supplementary concept word, protocol supplementary concept word, rare disease supplementary concept word, unique identifier, synonyms]

14. mutism.mp. [mp = title, abstract, original title, name of substance word, subject heading word, floating sub-heading word, keyword heading word, organism supplementary concept word, protocol supplementary concept word, rare disease supplementary concept word, unique identifier, synonyms]

15. schizophrenia.mp. [mp = title, abstract, original title, name of substance word, subject heading word, floating sub-heading word, keyword heading word, organism supplementary concept word, protocol supplementary concept word, rare disease supplementary concept word, unique identifier, synonyms]

16. movement dis*.mp. [mp = title, abstract, original title, name of substance word, subject heading word, floating sub-heading word, keyword heading word, organism supplementary concept word, protocol supplementary concept word, rare disease supplementary concept word, unique identifier, synonyms]

17. reactive attachment dis*.mp. [mp = title, abstract, original title, name of substance word, subject heading word, floating sub-heading word, keyword heading word, organism supplementary concept word, protocol supplementary concept word, rare disease supplementary concept word, unique identifier, synonyms]

18. (Down syndrome or trisomy 21).mp. [mp = title, abstract, original title, name of substance word, subject heading word, floating sub-heading word, keyword heading word, organism supplementary concept word, protocol supplementary concept word, rare disease supplementary concept word, unique identifier, synonyms]

19. exp Down syndrome/

20. exp attention deficit disorder/

21. exp motor dysfunction/

22. exp developmental disorder/

23. exp intellectual impairment/

24. conduct dis*.mp. [mp = title, abstract, original title, name of substance word, subject heading word, floating sub-heading word, keyword heading word, organism supplementary concept word, protocol supplementary concept word, rare disease supplementary concept word, unique identifier, synonyms]

25. exp tic/

26. cerebral palsy.mp. [mp = title, abstract, original title, name of substance word, subject heading word, floating sub-heading word, keyword heading word, organism supplementary concept word, protocol supplementary concept word, rare disease supplementary concept word, unique identifier, synonyms]

27. exp cerebral palsy/

28. 1 or 2 or 3 or 4 or 5 or 6 or 7 or 8 or 9 or 10 or 11 or 12 or 13 or 14 or 15 or 16 or 17 or 18 or 19 or 20 or 21 or 22 or 23 or 24 or 25 or 26 or 27

29. exp loneliness/

30. lonel*.mp. [mp = title, abstract, original title, name of substance word, subject heading word, floating sub-heading word, keyword heading word, organism supplementary concept word, protocol supplementary concept word, rare disease supplementary concept word, unique identifier, synonyms]

31. exp social isolation/

32. social* isolat*.mp. [mp = title, abstract, original title, name of substance word, subject heading word, floating sub-heading word, keyword heading word, organism supplementary concept word, protocol supplementary concept word, rare disease supplementary concept word, unique identifier, synonyms]

33. social* participat*.mp. [mp = title, abstract, original title, name of substance word, subject heading word, floating sub-heading word, keyword heading word, organism supplementary concept word, protocol supplementary concept word, rare disease supplementary concept word, unique identifier, synonyms]

34. exp social participation/

35. 29 or 30 or 31 or 32 or 33 or 34

36. 28 and 35

37. limit 36 to (infant or child or preschool child <1 to 6 years> or school child <7 to 12 years> or adolescent <13 to 17 years>)

PsychInfo:

S44	S9 AND S43	Limiters-Age Groups: Childhood (birth-12 yrs), Adolescence (13–17 yrs)	Interface-EBSCOhost Research Databases
		Search modes-Boolean/Phrase	Search Screen -Advanced Search
			Database - PsycINFO
S43	(S1 OR S2 OR S10 OR S11 OR S12 OR S13 OR S14 OR S15 OR S16 OR S17 OR S18 OR S19 OR S20 OR S21 OR S22 OR S23 OR S24 OR S25 OR S26 OR S27 OR S28 OR S29 OR S30 OR S31 OR S32 OR S33 OR S34 OR S35 OR S36 OR S37 OR S38 OR S39 OR S40 OR S41 OR S42)	Search modes-Boolean/Phrase	Interface - EBSCOhost Research Databases
			Search Screen -Advanced Search
			Database-PsycINFO
S42	DE “Cerebral Palsy”	Search modes-Boolean/Phrase	Interface-EBSCOhost Research Databases
			Search Screen -Advanced Search
			Database-PsycINFO
S41	cerebral palsy	Search modes-Boolean/Phrase	Interface-EBSCOhost Research Databases
			Search Screen-Advanced Search
			Database-PsycINFO
S40	down syndrome or trisomy 21	Search modes - Boolean/Phrase	Interface-EBSCOhost Research Databases
			Search Screen-Advanced Search
			Database-PsycINFO
S39	DE “Down’s Syndrome”	Search modes - Boolean/Phrase	Interface-EBSCOhost Research Databases
			Search Screen-Advanced Search
			Database-PsycINFO
S38	movement dis*	Search modes - Boolean/Phrase	Interface-EBSCOhost Research Databases
			Search Screen-Advanced Search
			Database-PsycINFO
S37	DE “Movement Disorders”	Search modes - Boolean/Phrase	Interface-EBSCOhost Research Databases
			Search Screen-Advanced Search
			Database-PsycINFO
S36	Schizophrenia	Search modes - Boolean/Phrase	Interface-EBSCOhost Research Databases
			Search Screen-Advanced Search
			Database-PsycINFO
S35	DE “Schizophrenia”	Search modes-Boolean/Phrase	Interface-EBSCOhost Research Databases
			Search Screen-Advanced Search
			Database-PsycINFO
S34	Mutism	Search modes-Boolean/Phrase	Interface-EBSCOhost Research Databases
			Search Screen-Advanced Search
			Database-PsycINFO
S33	DE “Mutism”	Search modes-Boolean/Phrase	Interface-EBSCOhost Research Databases
			Search Screen-Advanced Search
			Database-PsycINFO
S32	Tourette OR Tic dis*	Search modes-Boolean/Phrase	Interface-EBSCOhost Research Databases
			Search Screen-Advanced Search
			Database-PsycINFO
S31	DE “Tourette Syndrome” OR DE “Tics”	Search modes-Boolean/Phrase	Interface-EBSCOhost Research Databases
			Search Screen-Advanced Search
			Database-PsycINFO
S30	reactive attachment dis*	Search modes-Boolean/Phrase	Interface-EBSCOhost Research Databases
			Search Screen-Advanced Search
			Database-PsycINFO
S29	motor skills dis*	Search modes-Boolean/Phrase	Interface-EBSCOhost Research Databases
			Search Screen-Advanced Search
			Database-PsycINFO
S28	DE “Motor Skills” OR DE “Movement Disorders”	Search modes-Boolean/Phrase	Interface-EBSCOhost Research Databases
			Search Screen -Advanced Search
			Database-PsycINFO
S27	dyslexia OR acalculia OR agraphia	Search modes-Boolean/Phrase	Interface-EBSCOhost Research Databases
			Search Screen -Advanced Search
			Database-PsycINFO
S26	learning dis*	Search modes-Boolean/Phrase	Interface-EBSCOhost Research Databases
			Search Screen-Advanced Search
			Database-PsycINFO
S25	DE “Learning Disabilities” OR DE “Learning Disorders” OR DE “Dyslexia” OR DE “Acalculia” OR DE “Agraphia”	Search modes-Boolean/Phrase	Interface-EBSCOhost Research Databases
			Search Screen-Advanced Search
			Database-PsycINFO
S24	development* dis*	Search modes-Boolean/Phrase	Interface-EBSCOhost Research Databases
			Search Screen-Advanced Search
			Database-PsycINFO
S23	intellectual dis*	Search modes-Boolean/Phrase	Interface-EBSCOhost Research Databases
			Search Screen-Advanced Search
			Database-PsycINFO
S22	communication dis*	Search modes - Boolean/Phrase	Interface-EBSCOhost Research Databases
			Search Screen-Advanced Search
			Database-PsycINFO
S21	DE “Communication Disorders” OR DE “Language Disorders” OR DE “Developmental Disabilities”	Search modes-Boolean/Phrase	Interface-EBSCOhost Research Databases
			Search Screen-Advanced Search
			Database-PsycINFO
S20	autism	Search modes-Boolean/Phrase	Interface-EBSCOhost Research Databases
			Search Screen-Advanced Search
			Database-PsycINFO
S19	DE “Autism Spectrum Disorders”	Search modes-Boolean/Phrase	Interface-EBSCOhost Research Databases
			Search Screen-Advanced Search
			Database-PsycINFO
S18	child development dis*	Search modes-Boolean/Phrase	Interface-EBSCOhost Research Databases
			Search Screen-Advanced Search
			Database-PsycINFO
S17	DE “Intellectual Development Disorder”	Search modes-Boolean/Phrase	Interface-EBSCOhost Research Databases
			Search Screen-Advanced Search
			Database-PsycINFO
S16	conduct dis*	Search modes - Boolean/Phrase	Interface-EBSCOhost Research Databases
			Search Screen-Advanced Search
			Database-PsycINFO
S15	DE “Conduct Disorder”	Search modes-Boolean/Phrase	Interface-EBSCOhost Research Databases
			Search Screen-Advanced Search
			Database-PsycINFO
S14	child behavio* dis*	Search modes-Boolean/Phrase	Interface-EBSCOhost Research Databases
			Search Screen-Advanced Search
			Database-PsycINFO
S13	attention deficit	Search modes-Boolean/Phrase	Interface-EBSCOhost Research Databases
			Search Screen-Advanced Search
			Database-PsycINFO
S12	DE “Attention Deficit Disorder with Hyperactivity” OR DE “Attention Deficit Disorder”	Search modes-Boolean/Phrase	Interface-EBSCOhost Research Databases
			Search Screen-Advanced Search
			Database-PsycINFO
S11	anxiety	Search modes-Boolean/Phrase	Interface-EBSCOhost Research Databases
			Search Screen-Advanced Search
			Database-PsycINFO
S10	DE “Anxiety” OR DE “Anxiety Disorders”	Search modes-Boolean/Phrase	Interface-EBSCOhost Research Databases
			Search Screen-Advanced Search
			Database-PsycINFO
S9	(S3 OR S4 OR S5 OR S6 OR S7 OR S8)	Search modes-Boolean/Phrase	Interface-EBSCOhost Research Databases
			Search Screen-Advanced Search
			Database-PsycINFO
S8	social* participat*	Search modes-Boolean/Phrase	Interface-EBSCOhost Research Databases
			Search Screen-Advanced Search
			Database-PsycINFO
S7	DE “Social Isolation” OR DE “Social Deprivation”	Search modes-Boolean/Phrase	Interface-EBSCOhost Research Databases
			Search Screen-Advanced Search
			Database-PsycINFO
S6	DE “Patient Seclusion”	Search modes-Boolean/Phrase	Interface-EBSCOhost Research Databases
			Search Screen-Advanced Search
			Database-PsycINFO
S5	social* isolat*	Search modes-Boolean/Phrase	Interface-EBSCOhost Research Databases
			Search Screen-Advanced Search
			Database-PsycINFO
S4	lonel*	Search modes-Boolean/Phrase	Interface-EBSCOhost Research Databases
			Search Screen-Advanced Search
			Database-PsycINFO
S3	DE “Loneliness”	Search modes-Boolean/Phrase	Interface-EBSCOhost Research Databases
			Search Screen-Advanced Search
			Database-PsycINFO
S2	Neurodevelopmental Disorders” OR Neurodevelopmental dis*	Search modes-Boolean/Phrase	Interface-EBSCOhost Research Databases
			Search Screen-Advanced Search
			Database-PsycINFO
S1	DE “Neurodevelopmental Disorders”	Search modes-Boolean/Phrase	Interface-EBSCOhost Research Databases
			Search Screen -Advanced Search
			Database-PsycINFO
Note: * search function.			
